# Advances of antimicrobial dressings loaded with antimicrobial agents in infected wounds

**DOI:** 10.3389/fbioe.2024.1431949

**Published:** 2024-08-02

**Authors:** Yifan Gou, Liwei Hu, Xuejuan Liao, Jing He, Fan Liu

**Affiliations:** ^1^ Department of Stomatology, North Sichuan Medical College, Nanchong, Sichuan, China; ^2^ State Key Laboratory of Oral Diseases and National Center for Stomatology and National Clinical Research Center for Oral Diseases West China Hospital of Stomatology, Sichuan University, Chengdu, Sichuan, China

**Keywords:** wound dressings, antimicrobials, infected wounds, wound healing, biomaterials

## Abstract

Wound healing is a complex process that is critical for maintaining the barrier function of the skin. However, when a large quantity of microorganisms invade damaged skin for an extended period, they can cause local and systemic inflammatory responses. If left untreated, this condition may lead to chronic infected wounds. Infected wounds significantly escalate wound management costs worldwide and impose a substantial burden on patients and healthcare systems. Recent clinical trial results suggest that the utilization of effective antimicrobial wound dressing could represent the simplest and most cost-effective strategy for treating infected wounds, but there has hitherto been no comprehensive evaluation reported on the efficacy of antimicrobial wound dressings in promoting wound healing. Therefore, this review aims to systematically summarize the various types of antimicrobial wound dressings and the current research on antimicrobial agents, thereby providing new insights for the innovative treatment of infected wounds.

## Graphical Abstract

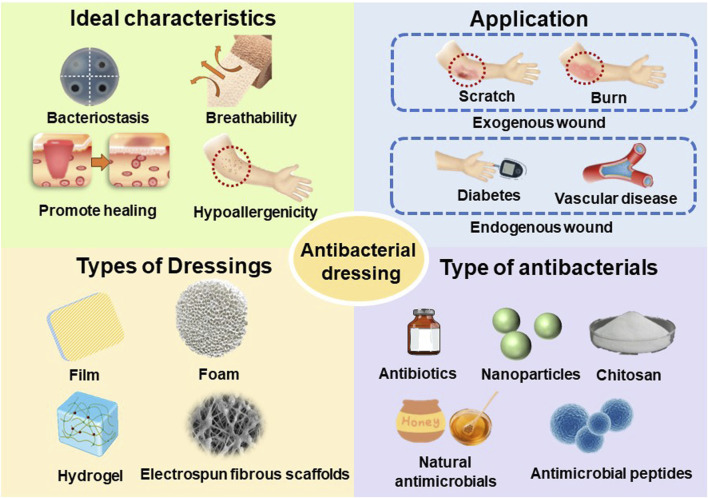

## 1 Introduction

The skin, as the body’s largest organ, covers the human body’s exterior, and it is essential for protecting the internal tissues, thermoregulation, sensation, immune function, and fluid balance (Nejaddehbashi et al., 2023). The proper functioning of the skin relies on the integrity of its tissue. (Hu et al., 2023), However, in daily life, the skin is highly susceptible to external environmental influences. Hundreds of millions of people worldwide suffer skin injuries every year due to external (abrasions, surgeries, and burns) or endogenous (diabetes mellitus and vascular disease) factors. (Kamoun et al., 2015). Studies have shown that in the United States alone, the expense of treating these wounds amounts to $25 billion annually. (Pang et al., 2023). Infected wounds can cause prolonged healing and increase the cost of wound management, resulting in significant personal and socio-economic burdens. Because of these significant financial and health costs, wound healing is a top research focus. In order to alleviate this costly burden, extensive research is being conducted with the aim of developing techniques that can effectively treat wounds (Homaeigohar and Boccaccini, 2020).

The dynamic process of wound healing begins when the skin is damaged and results in the restoration of the damaged skin. Wound healing is an intricate and organized biological process that may be categorized into four distinct and recurring phases: hemostasis, inflammation, proliferation, and remodeling (Tu et al., 2021) (Figure 2). These four phases involve interactions between multiple cell populations, soluble mediators, cytokines (Liang et al., 2022). Wounds are typically classified as either acute or chronic wounds, depending on the time it takes for them to heal. Acute wounds can fully regenerate anatomical and functional tissues within 3 weeks, while chronic wounds take up to 3 months to heal once they are formed. Bacterial infection is a significant factor in the development of chronic wounds. (Mo et al., 2022). The risk of wound infection is influenced by the interaction of three key factors: 1) host resistance to microbial interference; 2) local wound environment; 3) microbial bioburden (number, virulence and phenotype) (Hurlow and Bowler, 2022). Bacterial infection not only hampers the wound healing process, but also leads to significant tissue and cellular harm, potentially even endangering one’s life. Consequently, in the case of infected wounds, it is crucial to employ more sophisticated therapies and dressings to expedite wound healing and avoid infections. (DART and KINGSHOTT, 2019). A significant method of managing infected wounds is the creation of highly efficient wound dressings. (Sood et al., 2014).

**FIGURE 1:**
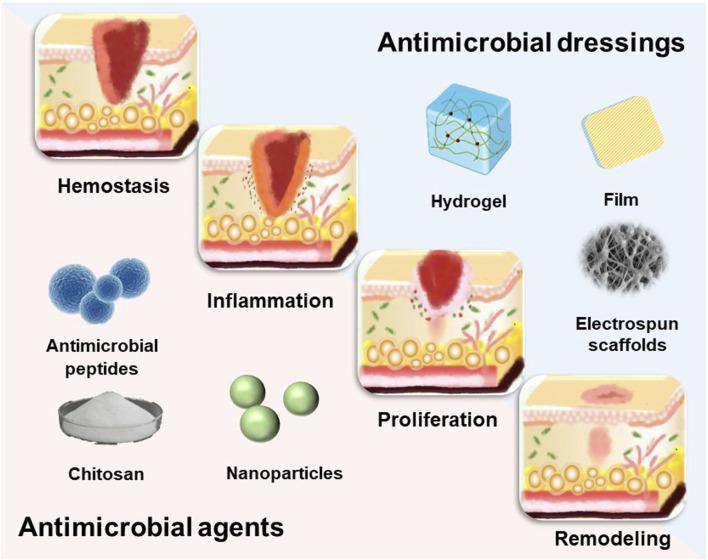
The dynamic process of wound healing.

**FIGURE 2:**
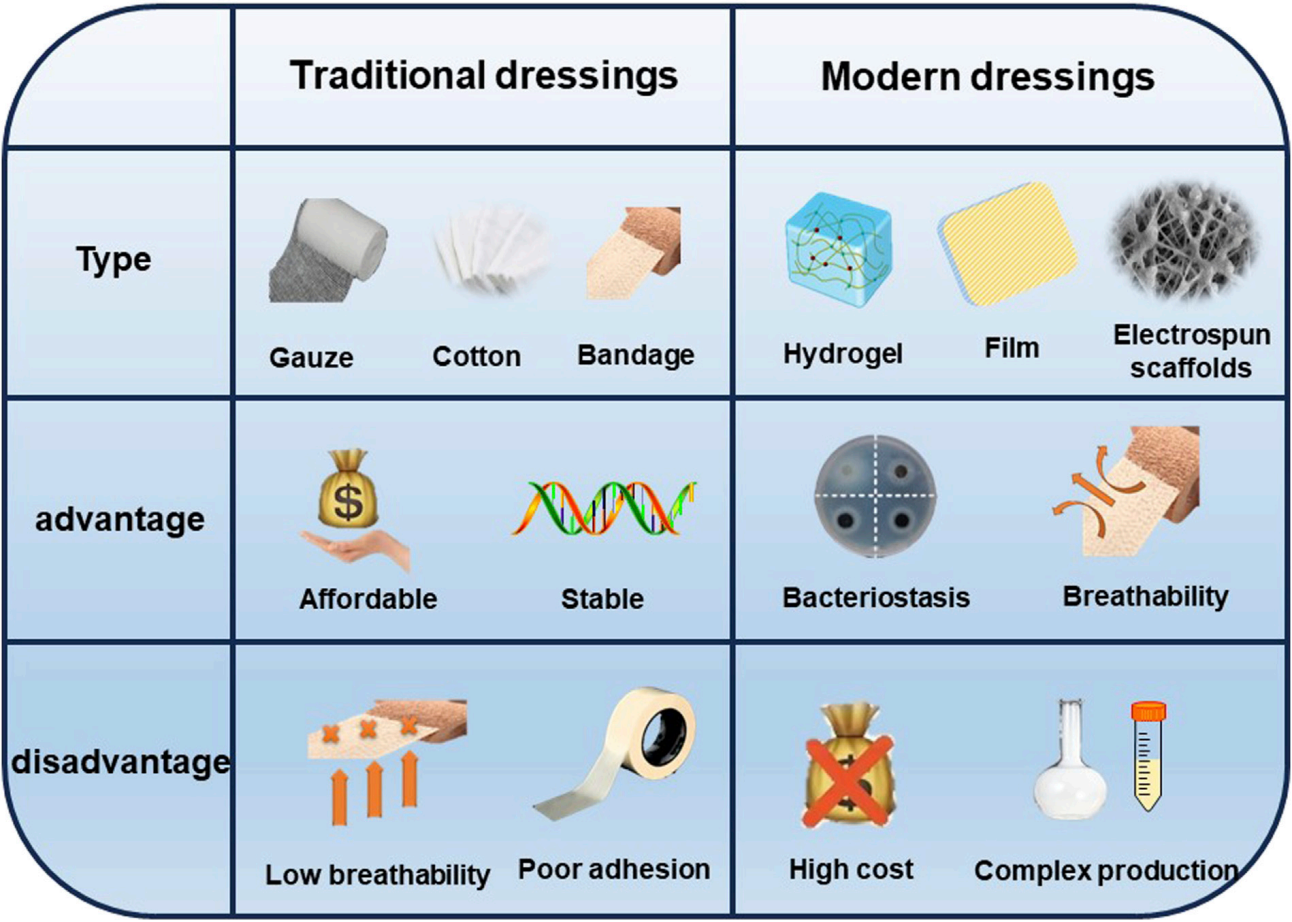
Comparison between traditional dressings and modern dressings.

**FIGURE 3:**
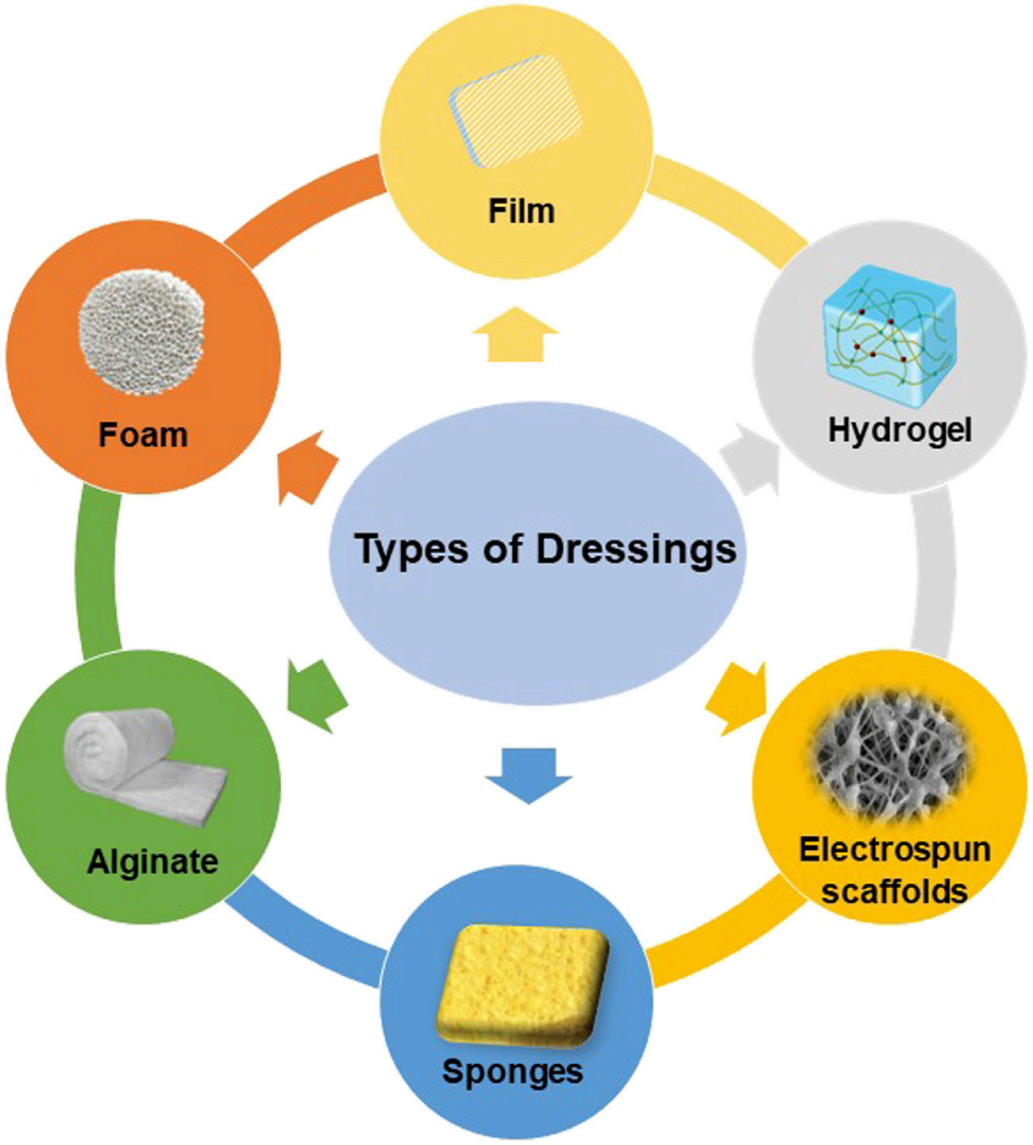
Classification of modifiable antimicrobial dressings.

Wound dressings serve the purpose of shielding wounds, eliminating exudate, preventing the infiltration of external bacteria, and enhancing the visual aspect of the wound. Traditional dressings (gauze, sterilized absorbent cotton, and bandages) commonly used in current clinical practice are economical; nevertheless, these materials possess many limitations, including insufficient osmotic absorption, inadequate wound surface adhesion, and limited permeability. The most promising wound dressings should be biocompatible, highly porous, and breathable, while preventing microbial invasion as well as driving epithelial formation. In addition, moist wound dressings are helpful to wound healing. So far, under the guidance of the concept of moist therapy, moist wound dressings mainly include film, foam, hydrocolloid, alginate and hydrogel (Homaeigohar and Boccaccini, 2020). However, simple moisturizing dressings cannot inhibit the bacterial growth process, in which case the bacterial infection may lead the wound into a chronic inflammatory state due to the prolonged inflammatory period, and the wound may become chronic or non-healing without an effective therapeutic strategy (Mandla et al., 2018) (Figure 3). Hence, the prompt administration of potent antimicrobial medicines is crucial in order to facilitate the process of wound healing. A dressing that combines antimicrobial agents can not only provide regulated antimicrobial treatment, but also enhance the extracellular matrix (ECM) to enhance cellular activity and facilitate wound healing. Nevertheless, there is currently no singular dressing that possesses all the necessary attributes to expedite the process of wound closure. (Mironczuk-Chodakowska et al., 2018). This review will discuss research advances in incorporating different types of dressing materials as well as antimicrobial agents to accelerate wound healing in hopes of providing new approaches to wound treatment.

## 2 Classification of modifiable antimicrobial dressings

**FIGURE 4:**
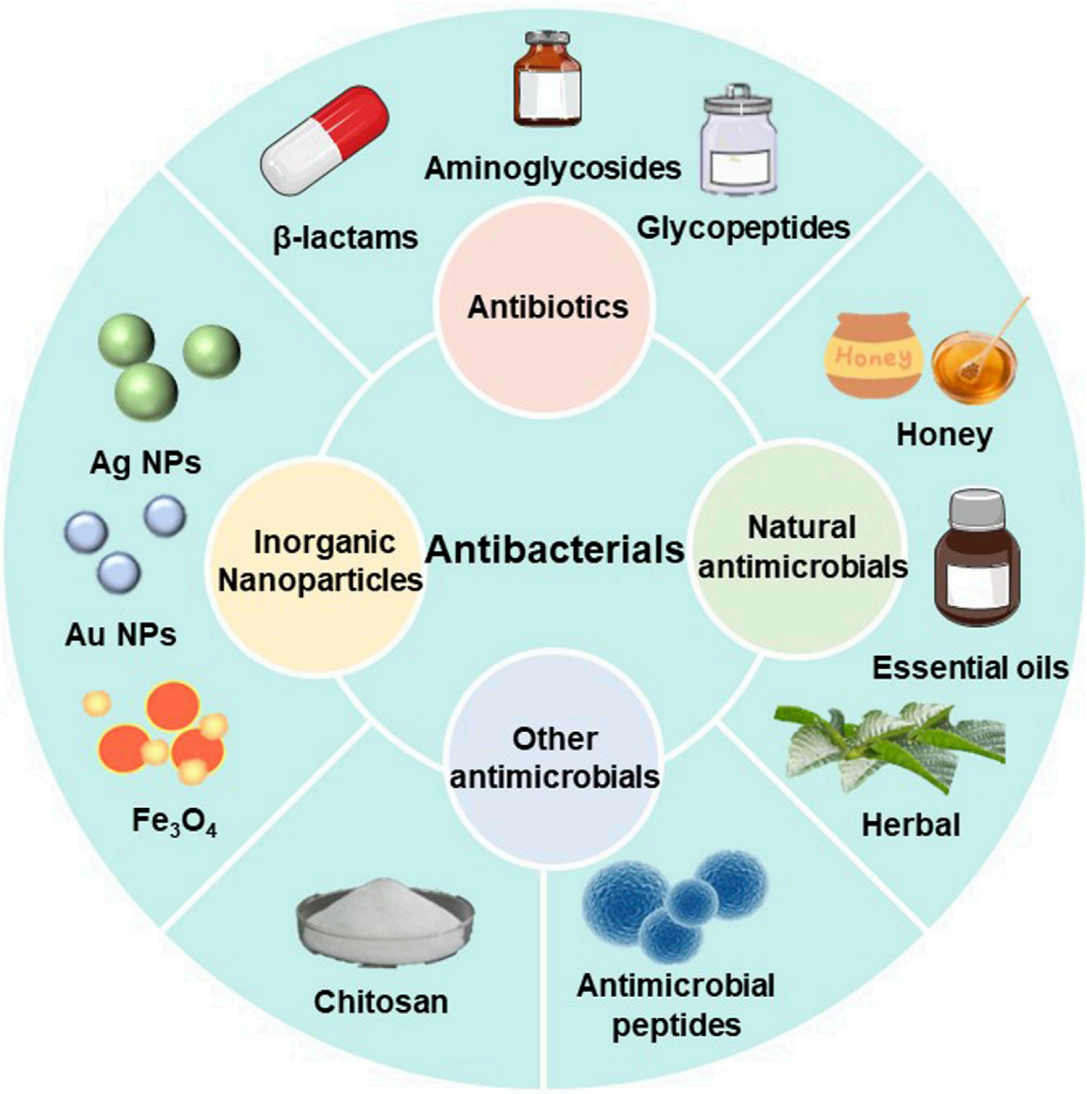
Classification of antimicrobial agents.

Ideal wound dressings should include properties such as biocompatibility, stability, breathability and hypoallergenicity, as well as the capacity to soak up exudates from the wound location and stimulate the regeneration of tissue. (Dhivya et al., 2015). The choice of biomaterials is crucial in effectively creating appropriate wound dressings. Various types of wound dressings have been developed to shield wounds from contamination and expedite the healing process. (Negut et al., 2018). These include films, foams, hydrogels, alginates, electrospun fibrous scaffolds, and other forms of dressings. (Figure 4).

### 2.1 Film

Films are translucent and elastic polymers with an adhesive that are frequently employed for superficial lacerations and mild exudative wounds (Powers et al., 2013). Films limit the penetration of fluids and microorganisms, but allow the passage of air and water vapor, thus shielding the wound from bacterial infiltration and the surrounding environment. Due to the limited absorption capacity of the film, insufficient fluid removal may occur, it may cause damage to newly formed epithelial cells. Single layer films are usually composed of a single polymer, mainly including materials such as polyethylene, polylactic acid, polyurethane, etc. Based on conventional film materials, it is now possible to prepare freestanding multilayer films using the layer-by-layer process, which involves the successive adsorption of polyelectrolytes with opposite charges onto a substrate. (Silva et al., 2015). The main advantage of freestanding multilayer films based on polyelectrolyte multilayer coatings over other types of conventional wound dressings is that The film permits the integration of various polyelectrolytes, bioactives, or particulate materials, either in combination or in distinct sections. As a result, a wide range of functions associated with wound dressings can be achieved, such as controlling swelling and permeability, promoting angiogenesis and thus promoting wound healing, as well as antioxidant and antibacterial functions. Adrian et al. used a layer-by-layer technique to prepare freestanding multilayered films composed of chitosan and alginate as opposing polyelectrolytes in an alternating manner. It was shown that they promote fibroblast migration and growth, which are promising wound dressings (Hautmann et al., 2022).

### 2.2 Foam

Foams typically comprise a dual-layered dressing, with an exterior layer that is permeable to water and repels it, and an inside layer that is in direct contact with the wound and attracts water. Foams are moderately absorbent, insulating, and are mostly used for moderately to highly exuding wounds. The foam dressing has sufficient adhesion and minimizes trauma during dressing changes. The foam dressings now commonly used in clinical practice also contain a permeable polyurethane film. At present, the prepolymer foaming process is mainly used in the market to produce medical polyurethane foam materials. The raw materials are polyisocyanates, polyols, foaming agents, surfactants, crosslinking agents and catalysts. Polyurethane foams possess excellent biocompatibility, appropriate flexibility, softness, minimal cytotoxicity, and satisfactory mechanical qualities. They are extensively employed as wound dressings, even when fully submerged in water, and are also more cost-effective than alternative treatments made from natural polymers. (Pahlevanneshan et al., 2021). Polyurethane foam dressings can also change their surface morphology to constitute a porous surface, and the porous structure gives them the advantage of superabsorbent capacity as well as the promotion of inward cellular growth to retain exudate and accelerate the healing process, preventing further wound infection (Liu et al., 2017). In addition, polyurethanes have the ability to be mixed with various fillers in order to create polyurethane composite foams that possess distinct characteristics. For example, Oryan et al. have shown that the use of polyurethane in combination with propolis has specific antimicrobial activity and can inhibit biofilm formation, enabling it to successfully fight different bacteria (Oryan et al., 2018).

### 2.3 Hydrogel

Hydrogels are three-dimensional structures made up of hydrophilic polymers that are crosslinked through physical or chemical methods. (Baranov et al., 2021). The properties of hydrogels obtained from different polymer materials and different preparation methods will be different, which can meet the application requirements in different fields. They serve as reservoirs for drugs and create microenvironments for therapy. These hydrogels can be modified by physical factors such as temperature, light, or magnetic fields, as well as chemical factors like pH or ionic strength. This allows them to effectively deliver drugs or provide sustained release of medications. (Lee et al., 2023). Hydrogels, due to their resemblance to the extracellular matrix, are biocompatible and can assist in maintaining moisture in drying wounds (Zhong et al., 2024). This promotes the development of new sarcomeres and creates a favorable environment for cell survival and division. Furthermore, hydrogels wooding dressings have various modes of adhesion. This kind of wound dressings have the capability to adhere to tissue interfaces either by physical means or by reacting with reactive groups in natural tissue proteins, thus achieving chemical adhesion through compositional design. This property allows the dressing to effectively seal the wound, expedite the process of hemostasis, and monitor the wound without the need to remove the dressing. (Fan et al., 2023). Hydrogels with the ability to transfer nutrients and oxygen provide new ideas for the preparation of antimicrobial dressings for dry, necrotic wound healing (Ying et al., 2019). A few studies have turned hydrogels into bilayer dosage forms after drying, giving them the ability to load multiple drugs (Xu et al., 2018). The design and development of injectable hydrogels that can be injected has further expanded the application of hydrogel dressings, which is advantageous for the management of wounds with uneven shapes (Xu et al., 2019). The investigation of innovative antimicrobial compounds and advancements in polymer science have facilitated the creation and refinement of antimicrobial hydrogels. These hydrogels have been increased in their ability to combat microbes by processes such as self-assembly, modification, and doping. (Jia et al., 2023). There are also studies using food-borne substances coupled with hydrogels to promote wound closure (Fang et al., 2023).

Alginate is a kind of natural polysaccharide polymer from brown algae seafood, its ions can be cross-linked to hydrogels. Hydrogels prepared by alginate can be divided into physical gels and chemical covalent gels according to their formation mechanism. This kind of gel has stable mechanical properties and high biocompatibility, is an excellent hemostatic material, and is widely used in the field of biomedicine. Alginate wound dressings are non-woven fabric and non-adhesive fiber made from calcium and sodium salts of alginate, mostly used for highly exudative wounds. The dressings are highly absorbent, nonadherent, biodegradable, and may contain controlled-release ionized silver to prevent microbial contamination (Sood et al., 2014). It is important to note that alginate dressings are contraindicated in dry wounds as they promote absorption but not hydration. And alginate dressings should be changed regularly especially in infected wounds. The physical characteristics, ability to form a gel, and strength of alginate gels are influenced by various factors including the distribution of molecular weights, the composition of glyoxylate, the relative proportions of the three blocks, the source of calcium ions, and the technique of preparation. (Sosa-Herrera et al., 2012). One effective method to improve alginate dressings is by incorporating inorganic or organic enhancements to create polysaccharides and nanocomposites with varying molecular weights and specialized structures (such as porous and caged structures). This enhances the mechanical properties of the dressings and allows for a higher adsorption capacity. (Jiao et al., 2019).

### 2.4 Electrospun fibrous scaffolds

Electrostatic spinning is an uncomplicated and inexpensive technique for fabricating fiber matrices capable of generating fibers within the nanometer to micrometer size range. Electrospinning technology is the only method that can directly and continuously prepare polymer nanofibers. The fibers prepared by it have a certain three-dimensional structure, which not only provides a favorable space for cells to obtain nutrition, growth, and metabolism. The fibrous scaffolds possess a shape that closely resembles the natural extracellular matrix, hence enhancing cell attachment, proliferation, migration, and gene expression features. (Sill and von Recum, 2008). Electrostatically spun fibers are often on the nanoscale and possess significant utility in diverse domains owing to their diminutive dimensions, expansive specific surface area, elevated porosity, substantial aspect ratio, and consistent and steady homogeneity. Nanofibers produced through the process of electrostatic spinning using biodegradable filaments have been utilized to create nanofibrous membranes. These membranes possess a structure similar to the extracellular matrix, serving as a framework for the growth of skin cells and promoting tissue regeneration. (Yang et al., 2018; Ji et al., 2022). Electrostatically spun nanofibers possess a high specific surface area, high porosity, and tiny size, allowing for the loading of pharmaceuticals using multiple processes (Akhmetova and Heinz, 2020), such as antibiotics can be easily loaded into nanofibers by hybrid electrostatic spinning, and they can facilitate the exchange of air at the wound site and maintain a wet healing environment. (Unnithan et al., 2012). In addition, electrospun 3D scaffolds often possess a well-connected porous structure and a large surface area, which offer distinct benefits in the treatment of persistent wounds. The high porosity of this material enables efficient nutrition and oxygen transmission, as well as effective removal of metabolic wastes, improving cell viability and proliferation. It has been shown that treatment with fibrous scaffolds accelerated the closure of chronic wounds within 14 days (Chen et al., 2016).

### 2.5 Other polymeric wound dressings

Other types of polymeric wound dressings, including sponges (Yang et al., 2024), bandages (Wu et al., 2022), hydrocolloids (Tang et al., 2023), collagen (Brooker and Tronci, 2023), and others.

Sponges are pliable and resilient wound dressings that possess a porous structure, allowing them to effectively absorb substantial quantities of wound exudate. Due to their hydrophilicity and ability to interact with cells, sponge dressings are commonly used as both hemostatic agents and regenerative materials for burn burns. However, sponge substitutes still suffer from weak mechanical strength, insufficient antimicrobial properties, and easy impregnation of wounds in practical applications (Alven et al., 2022). Furthermore, bandages are dressings for wounds that possess comparable characteristics to sponges. They have the ability to be enclosed with diverse bioactive substances to aid in the management of wound care (Alven et al., 2022).

Hydrocolloid dressings are made up of hydrophilic and self-adhesive colloidal particles that are covered with a waterproof polyurethane coating on the outside. Colloidal particles typically consist of gelatin, pectin, and carboxymethyl cellulose (CMC) and exhibit a range of forms, sizes, and thicknesses. (Nguyen et al., 2023). They provide a humid environment by combining with wound fluid to produce a gel and ensuring its permeability. However, hydrocolloids are impermeable and more durable but should not be used in exuding wounds due to their impermeability.

Collagen is a highly suitable option for utilization as a wound dressing in the realm of biomaterials. The type, amount and organization of collagen changes in healing wounds and determines the tensile strength of healing skin (Mathew-Steiner et al., 2021). It is crucial to confer antibacterial capabilities to collagen and enhance its physicochemical characteristics.

Wound dressings generally require excellent antimicrobial properties and biocompatibility, as well as non-invasive, painless separation when their function is completed. Compared with traditional medical wound dressings, new medical wound dressings developed by researchers in recent years can integrate a variety of excellent functions, achieving antibacterial, antioxidant, hemostatic, promote healing, reduce scar formation and other effects. However, how to integrate and utilize the antimicrobial properties, biocompatibility and mechanical properties of wound dressings to develop fully functional advanced wound dressings is still a great challenge for researchers.

## 3 Classification of antimicrobial agents

**FIGURE 5:**
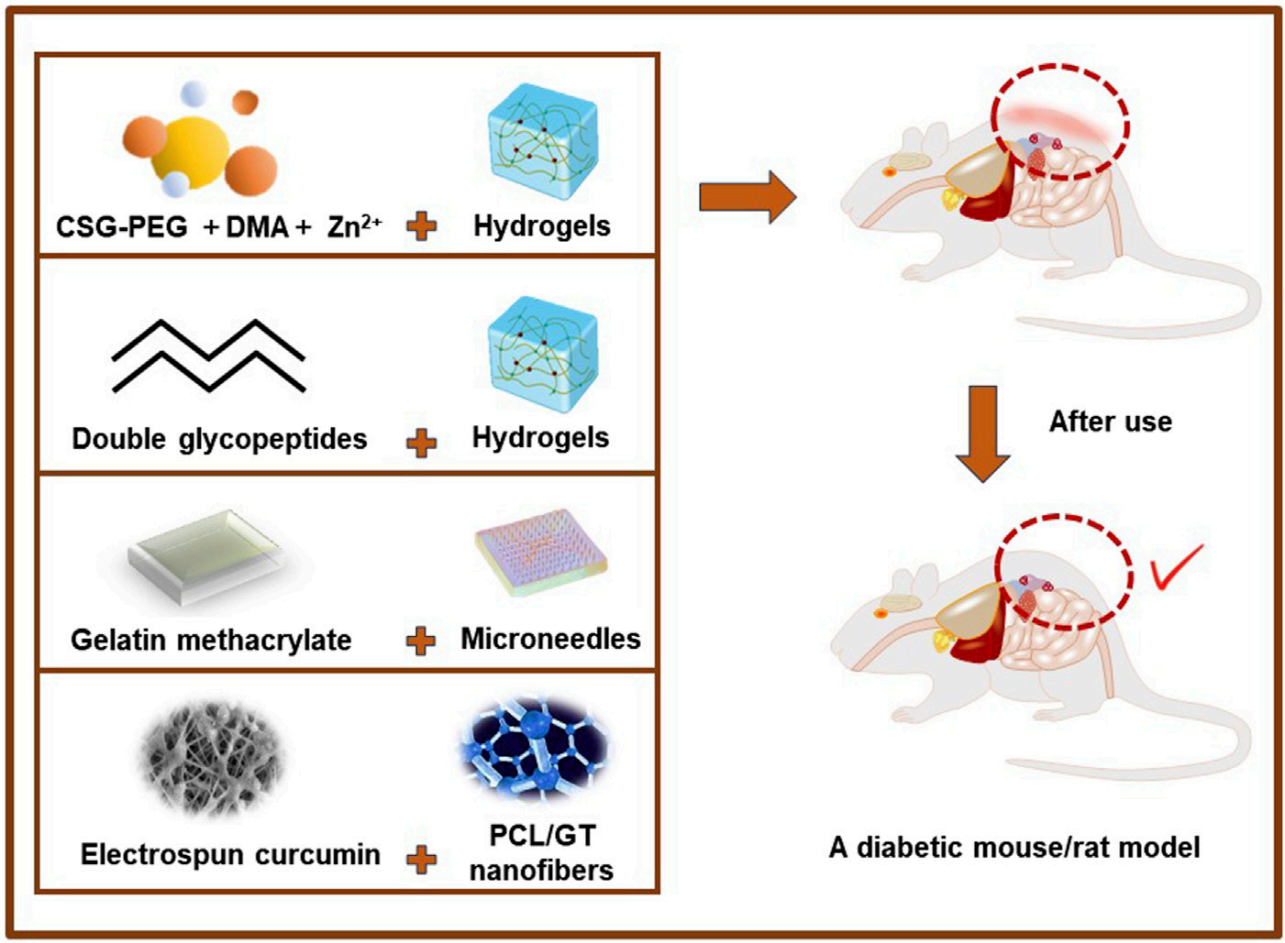
Application of partial antibacterial dressings in vivo experiments.

Chronic wounds are susceptible to bacteria and microorganisms, and when bacteria adhere to the wound surface they can lead to the formation of biofilms, which are bacteria-rich areas that protect bacteria and associated microorganisms from the immune system and antibiotics. Even though all types of wound dressings act as mechanical barriers to protect the wound tissue, wounds should be treated using wound dressings that contain antimicrobial agents in order to prevent bacterial infection and the production of biofilms in the wound environment. There is an extensive collection of material available on various antibacterial components used for wound healing, including antibiotics (Chen et al., 2023), nanoparticles (Luo et al., 2023), natural antimicrobials (Bahari et al., 2022), antimicrobials based on natural polymers (Khosravimelal et al., 2021; Yaxian Liang et al., 2023) and others(Figure 5).

### 3.1 Antibiotics

Antibiotics are the predominant and highly efficient antibacterial agents. (Norowski and Bumgardner, 2008). Antibiotics have been of great interest as dressing additives. Although antibiotics have antibacterial properties and the ability to promote wound healing, their excessive usage can result in the development of bacterial resistance. (Yang et al., 2020). Recently, topical antibiotic administration has received increasing attention, and the direct use of antibiotics in wound dressings can provide sufficient local bactericidal dose directly to the site of infection, and can control the local concentration and sustained release, as well as reduce the risk of antibiotic resistance (De Giglio et al., 2011). In the case of chronic wounds like diabetic foot ulcers (Fang et al., 2024), it is more effective to apply antibiotics directly to the affected area for a short duration, rather than administering them across the entire body, because the blood flow in the extremities is not very good. Therefore, topical administration with wound dressings is more effective and selective for wounds with minimal side effects (Homaeigohar and Boccaccini, 2020). Despite the variety and number of antibiotics that have been developed, few of them are used for clinical applications due to toxic effects and difficult cellular uptake. To date, the only antibiotics available for use are aminoglycosides, β-lactams, glycopeptides, quinolones, sulfonamides, and tetracyclines (Negut et al., 2018; Yang et al., 2020).

Among them, ciprofloxacin (CIP) is a very popular antibiotic with an antimicrobial spectrum that includes both Gram-positive and Gram-negative bacteria (Ma et al., 2016). Studies have demonstrated that hydrogels containing ciprofloxacin have significant promise for therapeutic use, particularly in the treatment of infectious disorders. These hydrogels exhibit strong antibacterial characteristics and provide sustained benefits. (Das and Pal, 2015). In addition, gentamicin is a traditional broad-spectrum antibiotic (Alzarea et al., 2022) used for infections. But the drug’s systemic toxicity, particularly towards the kidneys, and its low plasma concentrations have impeded its usage. Nevertheless, the use of topical administration overcomes the constraints associated with the use of gentamicin.3.2 Nanoparticles (Yang et al., 2018)

### 3.2 Inorganic nanoparticles

Nanomaterials encompass a diverse range of antimicrobial compounds, which can be classified into many categories, including metal-based and carbon-based variants. Their physicochemical qualities and dimensions make them versatile for tissue regeneration and wound healing. They possess antibacterial and anti-inflammatory activities, enhance intercellular connections, and promote stem cell differentiation. (Norahan et al., 2023). Among numerous particle antimicrobial agents, metal ions are mild and efficient in their antimicrobial activity (Mouriño et al., 2011), effectively sterilizing the bacteria while avoiding the burns that may be caused by photothermal antimicrobial methods (Yang et al., 2022).

Silver nanoparticles (Ag NPs) can be produced using several methods, such as physical, chemical, and biological approaches (Liu et al., 2023). Because of their exceptional antibacterial characteristics, nanoparticles derived from them have been extensively employed in the production of cosmetics. (Liao et al., 2019). The prevailing consensus among scholars is that Ag NPs engage with cysteines in specific protein areas on the bacterial membrane, resulting in the loss of internal K and the disruption of cellular transport systems. This finally culminates in the demise of bacterial cells. Gold nanoparticles (Au NPs) can bind to the bacterial membrane, causing the release or entry of bacterial contents into the outer membrane and peptidoglycan layer. This ultimately leads to the death of the bacteria. (Yang et al., 2018).

Besides Ag NPs and Au NPs, there are several additional metal nanoparticles that exhibit antibacterial properties, including iron oxide (Fe3O4), titanium oxide (TiO2), and zinc oxide (ZnO). For example, Cai et al. (2016) developed chitosan/gelatin hybrid nanofibers, which enhanced the antimicrobial properties of nanofibers by adding Fe_3_O_4_ particles. Titanium oxide nanoparticles demonstrate bactericidal activity upon exposure to UV irradiation (Homaeigohar and Boccaccini, 2020). Zn is a crucial element that impacts cellular function. It plays a role in the production of DNA and RNA, which are essential for cell replication, differentiation, and transcription. Zn ions can induce bacterial mortality by impeding active transport, amino acid metabolism, and altering enzyme systems. (Sirelkhatim et al., 2015).

### 3.3 Natural antimicrobial agents

Honey is a therapeutic nutrient that include notable antibacterial, anti-inflammatory, and antioxidant effects. (Pleeging et al., 2022). The antimicrobial and bactericidal activity of honey can be attributed to several factors: 1) High sugar content and high osmotic pressure of honey, which reduces water activity. High osmotic pressure hinders the development of microorganisms (Molan, 2016). 2) It has an acidic pH (usually in the range of 3.4–6.1), the acidity of honey may promote bacterial eradication by macrophages and inhibit the establishment of microbial biofilms, and it is also capable of acidifying alkaline mediators in chronic wounds, thus stimulating wound healing (Al-Waili et al., 2011).3) Presence of antimicrobial components such as the production of peroxides, nitric oxide and prostaglandins (Boateng and Diunase, 2015).

Essential oils (Wang et al., 2021) possess a range of beneficial properties such as antiviral, antioxidant, insecticidal, anticancer, antiallergic, anti-inflammatory, and antimicrobial effects. They are synthesized from various parts of plants and are considered secondary metabolites. These oils have been successfully combined with biopolymer nanofibers to create wound dressings for medical applications. The antibacterial properties of essential oils are attributed to their phenolic constituents, particularly thymol and carvacrol (Homaeigohar and Boccaccini, 2020). Wang et al. used eucalyptus essential oil (EEO), ginger essential oil (GEO) and cumin essential oil (CEO) to prepare carboxymethyl chitosan (CMC) and carbomer 940 (CBM) physically crosslinked effective antibacterial hydrogel. The results show that CBM/CMC/EEO hydrogel can accelerate wound healing in mouse burn model by promoting the recovery of dermis and epidermis (Wang et al., 2021).

Herbal extracts have also attracted widespread attention, and the combination of herbal extracts with biopolymer nanofibers is expected to be used (Homaeigohar and Boccaccini, 2020). However, the therapeutic efficacy of herbal extracts is constrained by several variables, such as the absence of targeted delivery and suboptimal absorption. (Yang et al., 2018). Ahn et al. use alfalfa, an ancient medicinal plant that contains antibacterial/oxygenating chlorophylls and bioactive phytoestrogens, as research subjects. These findings demonstrate alfalfa-based nanofibers promote wounds healing by promoting re-epithelialization and granulation tissue formation in both mouse and human skin. (Ahn et al., 2019).

Polyphenols are essentially secondary metabolites of polyphenol structures. Polyphenols are most commonly found in plant-based foods, such as tea and coffee beans, which are rich in polyphenols. (Tsao, 2010). Polyphenols have attracted widespread attention in recent years for their application prospects in wound repair due to their various biological activities such as antibacterial and anti-inflammatory properties. Epigallocatechin gallate (EGCG) can form a hydrogel dressing with 3-acrylamido-phenylboronic acid. The EGCG released from this dressing not only has the effect of resisting oxidation, sterilization and promoting angiogenesis, but also can mobilize the transformation of macrophages to show anti-inflammatory effect, and ultimately has an ideal ability to promote healing in the model of diabetes mice with full-thickness cortical defect wounds (Zhao et al., 2021). Furthermore, the incorporation of tannic acid (TA), a polyphenolic molecule known for its antioxidant, anti-inflammatory, and antibacterial characteristics, into 3D sponges enhances their antimicrobial and antioxidant capabilities, eliminating the necessity for supplementary antibiotics. (Huang et al., 2023). And curcumin (Hu et al., 2021), for example, is derived from the spice turmeric, a yellow pigment with associated antibacterial, anti-inflammatory, antioxidant and anticancer properties.

### 3.4 Chitosan

Chitosan is a natural cationic polysaccharide consisting of (1→4)-2-amino-2-deoxy-β-D-glucan, a partially or completely deacetylated form of chitin (Xie et al., 2023). The physicochemical characteristics of chitosan play a crucial role in determining its functional capabilities. Chitosan can undergo chemical modifications, including as acylation, carboxylation, and grafting of other synthetic or natural compounds onto its macromolecular chain, in order to enhance its gel properties. One of the crucial features of chitosan is its degree of acetylation, which refers to the arrangement of amino groups along the polymer chain. The degree of acetylation directly influences the solubility, swelling rate, bioactivity, and biodegradation of chitosan, hence impacting its functional characteristics. Additionally, the molecular weight of chitosan is a significant physicochemical characteristic (Baranov et al., 2021). By controlling the molecular weight of chitosan, its viscosity can be reduced and its water solubility can be improved (Matica et al., 2019). Lu et al. (2023) crosslinked chitosan dissolved in alkaline solution to form a topological structure via 4-arm-PEG-CHO and 4-arm-PEG-NH. The macromolecular crosslinked chitosan hydrogels exhibited notable benefits in terms of their mechanical characteristics, antibacterial capabilities, and swelling properties, making them promising for use in dressings. Some scholars (Yang et al., 2022) boosted the water solubility and increased the mechanical strength of the hydrogels by modifying chitosan with polyethylene glycol monomethyl ether. Additionally, they observed outstanding biocompatibility. Chitosan has significant biocompatibility and biodegradability (Lozano-Navarro et al., 2017) low toxicity (Lu et al., 2017) and important biological properties such as antimicrobial and antioxidant (Chen et al., 2015).

### 3.5 Antimicrobial peptides

Antimicrobial materials usually suffer from insufficient efficiency or induction of cytotoxic effects. However, in the last few years, a new generation of antimicrobial agents called antimicrobial peptides (AMP) has emerged (Homaeigohar and Boccaccini, 2020; Kazemzadeh-Narbat et al., 2020). AMP consist of short sequences of cationic amino acids produced by most organisms. These cationic molecules may be attracted to negatively charged bacterial surfaces (Norahan et al., 2023), thereby altering the electrostatic charge on the bacterial surface and disrupting the bacterial membrane, which can lead to bacterial lysis (Ageitos et al., 2017). AMPs exhibit potent antimicrobial action and a wide antimicrobial range, effectively targeting both Gram-positive and Gram-negative bacteria, fungi, and viruses. (Yang et al., 2018). Although AMP still has drawbacks, such as tissue toxicity and hemolysis (Steckbeck et al., 2013), AMP demonstrates superior biocompatibility index values in comparison to synthesized medicines of similar structures, however, further studies are needed to investigate its feasibility as an antimicrobial agent due to the fact that AMP is unstable and prone to degradation.

### 3.6 Other antimicrobial agents

In addition to the antibacterial agents mentioned above, there are many other types of antimicrobial agents. Researchers have utilized ionic liquids (ILs) made up of organic cations and anions, taking inspiration from the natural raw material chitosan. ILs commonly disrupt bacterial membranes by inducing the formation of holes, leading to the fast leakage of cellular contents and subsequent cell death. (Jia et al., 2023). In addition to ionic liquids, polyethyleneimine (PEI) (Liu et al., 2021) is another antimicrobial polymer used in wound healing applications and contains repeating amine units that can be protonated at physiological PH value. The positive charges of these substances interact with the negatively charged surfaces of bacteria, resulting in the death of the bacteria through mechanisms such as damaging their membranes or causing cellular depolarization (Norahan et al., 2023). Dextran refers to a homotypic polysaccharide composed of glucose as the monosaccharide, and the glucose units are connected by glycosidic bonds. Dextran is widely used in medical products and serves as a component of drug delivery nanoparticles (NP). Hydrogel scaffolds with a high proportion of dextran promote rapid wound healing due to their overall physical properties (Sun et al., 2011). And hyaluronic acid (HA) has a variety of biological functions, such as promoting lubrication, reducing inflammation, and relieving pain. But an ongoing challenge for HA is its short residence time in the body (Gilpin et al., 2021). Last but not least, collagen plays an important role in the formation of the extracellular matrix (ECM) and the development of cells and tissues. Experiments have shown that collagen hydrogel wound dressings can significantly accelerate burn wound healing and new skin generation (Ge et al., 2020).

At present, antimicrobials still have some shortcomings: such as the abuse of traditional antibiotics and other antibacterial drugs, many drug-resistant bacteria have evolved. The development of antimicrobials with combined antimicrobials, the precise selection of antimicrobials according to the situation and the strict control of the timing and dosage of the release of antimicrobials will be the key to overcoming these shortcomings, thus providing a new approach to antimicrobial therapy.

## 4 Evaluation of wound dressings

### 4.1 *In vitro* experiments

Yang et al. introduced cationic polyelectrolyte brushes grafted from bacterial cellulose (BC) nanofibers into polydopamine/polyacrylamide hydrogels, and designed and prepared a new class of hydrogel dressings with high tensile, adhesive, biocompatible and antibacterial properties. The findings from the contact sterilization trials demonstrated a positive correlation between the BCD ratio and both the effectiveness and duration of antibacterial activity. (Yang et al., 2021). The positively charged quaternary ammonium group provides the hydrogel with long-lasting antibacterial properties and promotes the proliferation of negatively charged epidermal cells. In addition, hydrogels are rich in catechol groups and are able to adhere to a variety of surfaces. Therefore, the multi-functional hydrogel has stable coverage, durable antibacterial and rapid wound healing, which shows the prospect of wound dressing. Cheon et al. synthesized three types of silver nanoparticles (Ag NPs) in aqueous solution, namely, spherical (Ag NSs), discoidal (Ag NDs), and triangular platelet (Ag NTs). The study demonstrated that Ag NSs had the largest inhibitory zone, measuring 4.8 mm in width. In addition, all three types of Ag NPs showed high activity against *E. coli*, with the inhibitory activity in the order of Ag NSs > Ag NDs > Ag NTs. (Cheon et al., 2019). The reason for this difference in antimicrobial activity is that Ag NPs expresses a mechanism that destroys cell membranes, leading to cell death. However, in fibroblasts, Ag NPs is absorbed into the cell through endocytosis. Therefore, the antimicrobial activity can be controlled by controlling the shape and size of Ag NPs. There are also studies that formulated chitosan/polyvinyl alcohol (PVA)-based honey hydrogel films using solvent casting method and performed various tests such as *in vitro* drug release, antimicrobial studies, and stability tests to evaluate potential wound healing applications. The benefit is the development of antimicrobial dressings with antimicrobial effect without using any harmful organic chemicals or solvents (Chopra et al., 2022). Wang et al. processed cellulose acetate (CA) and polycaprolactone (PCL) as polymer carriers for silver nanoparticles (Ag NPs) and lavender oil (LO), respectively, to form a bilayer Janus fiber. (Ito et al., 2013). The polymer disrupts bacterial cell membranes and increases their permeability, and silver ions can enter the bacteria, inhibit respiration and lead to bacterial death (Alven et al., 2022; Wang et al., 2022). Hydrophobicity is an important function of wound dressings to prevent external water intake, which can cause infection and inflammation. All fibers in this experiment are highly hydrophobic and suitable for wound dressing applications, which will effectively protect wounds from infection. Mohandas et al. developed chitosan-hyaluronic acid composite sponges containing fibronectin nanoparticles and vascular endothelial growth factor (VEGF). *In vitro* release experiments showed that the VEGFs were initially released rapidly. The results indicated that the prepared chitosan-hyaluronic acid/VEGF loaded fibrin nanoparticles composite sponges (CHVFS) have the potential to induce wound healing angiogenesis (Mohandas et al., 2015; Yang et al., 2018).

### 4.2 *In vivo* experiments

Yang et al. developed photo-crosslinked multifunctional antibacterial adhesive anti-oxidant hemostatic hydrogel dressings based on polyethylene glycol monomethyl ether modified glycidyl methacrylate functionalized chitosan (CSG-PEG), methacrylamide dopamine (DMA) and zinc ion for the disinfection of drug-resistant bacteria and the promotion of wound healing (Ito et al., 2013). The study assessed the rate at which wounds closed, the thickness of granulation tissue, the amount of collagen deposition, and the regeneration of blood vessels and hair follicles in a mouse model with complete skin defects that were infected with *staphylococcus aureus* (MRSA). The findings demonstrated that these versatile antimicrobial adhesive hemostatic hydrogels exhibited superior effectiveness in promoting healing, indicating their potential as viable options for treating infected wounds. (Yang et al., 2022). There have also been studies on hybrid hydrogels assembled by double synthetic glycopeptides were designed to simulate extracellular matrix dressings for wound healing of bacterial infected skin without additional therapeutic agents or cells. MRSA-infected full-thickness wounds were established on the back of diabetic SD (Sprague Dawley) rats. This hydrogel accelerated wound healing and significantly improved epidermal regeneration compared to untreated or common hydrogel treated (Liu et al., 2022). Overall, ECM mimics and immunomodulatory glycopeptide hydrogels are promising multifunctional dressings that can reshape the damaged tissue environment without additional drugs, exogenous cytokines, or cells, providing an effective strategy for the repair and regeneration of chronic skin wounds. In addition, based on dynamic covalent bonds, photo-triggered covalent bonds and hydrogen bonds, multifunctional bio-adhesive hydrogels comprising modified carboxymethyl chitosan, modified sodium alginate and tannic acid are developed (Zou et al., 2022). This hydrogel shows excellent hemostatic performance and healing promoting ability in rabbit liver injury model, and has attractive application value in first-aid hemostasis and infected wound healing. Meng et al. reported that a gelatin methacrylate (GelMA) microneedle (MNs) patch that has unique biological properties, can penetrate the skin by injection without causing significant pain, and can maintain the bioactivity of epitopes and drugs *in vitro*. Using a diabetic mouse model, the results showed that MNs can effectively control drug release to improve cell migration function and accelerate collagen deposition, epithelial regeneration, and angiogenesis (Yuan et al., 2022). Therefore, the patch offers a potentially valuable method for repairing diabetic wounds in clinical applications. Choi et al. used electrostatic spinning to formulate PEG-PCL [poly (epsilon-caprolactone) (PCL) and poly (ethyleneglycol) (PEG)] hybrid nanofibers encapsulated with human epidermal growth factor (EGF) for the treatment of diabetic ulcers. A study was conducted on diabetic mice to evaluate the effectiveness of EGF-encapsulated nanofibers in closing wounds. The results showed that wounds treated with EGF-encapsulated nanofibers had better healing rates on the seventh day compared to wounds treated with plain nanofibers or EGF encapsulation alone. This study suggests that EGF-conjugated nanofibers can be used as a novel wound healing material by increasing the proliferation and phenotypic expression of keratinocytes (Choi et al., 2008; Ito et al., 2013). This study showed that EGF-conjugated nanofiber could potentially be employed as a novel wound healing material by increasing proliferation and phenotypic expression of keratinocytes.

Ranjbar-Mohammadi et al. describe the potential of electrospun curcumin-loaded poly (ε-caprolactone) (PCL)/gum tragacanth (GT) (PCL/GT/Cur) nanofibers for wound healing in diabetic rats. *In vivo* wound closure experiments showed that after 15 days, pathological studies indicated that the application of GT/PCL/Cur nanofibers resulted in significantly faster wound closure and significantly faster granulation tissue formation (Ranjbar-Mohammadi et al., 2016). Wu et al. reported the use of filipin-chitosan films encapsulated with adipose-derived stem cells (ADSC) for diabetic wound care. An *in vivo* wound healing study using diabetic SD rats demonstrated that wound tissue encapsulated in ADSCs-loaded filipin-chitosan films regenerated almost in the vicinity of near-normal tissue. (Wu et al., 2018) (Figure 6). These experiments all demonstrated that various types of antibacterial dressings had effective antibacterial properties, promoted vascular regeneration and regulated cell microenvironment, providing effective theoretical support for further clinical application.

### 4.3 Clinical application

Novel antimicrobial dressings have now been used clinically by some scholars to expand the efficacy and application of antimicrobial dressings in wound care. (Table 1).Silver-containing silicone foams reported by Tong et al. in a clinical study demonstrated that all diabetic ulcers of the patients significantly showed positive wound closure and reduction over a period of 3–16 weeks (Alven et al., 2022). And there were no clinical signs of infection at the end of the follow-up period (JW, 2009). Coutts et al. co-loaded polyvinyl alcohol (PVA) foam wound dressings with gentian violet and methylene blue on chronic wounds of the lower extremities in diabetic patients with bacterial infections. Upon the conclusion of the assessment period, particularly in diabetic foot ulcers, there was an improvement in surface critical colonization and pain scores. Furthermore, a decrease in the size of the wound was noted in 8 out of 14 patients (57%) by the fourth week. (COUTTS and SIBBALD, 2014). The well-known Rivelin formulation consists of Polyvinylpyrrolidone (PVP), Eudragit RS100 drug-carrying fibers and a polycaprolactone (PCL) backing film. Colley et al. prepared a mucoadhesive nanofiber film by combining PVP, Eudragit RS100 and Polyethylene oxide (PEO). This nanofiber film was then combined with a hydrophobic protective layer made from heat-treated PCL nanofibers to form a bilayer membrane system (Yang et al., 2018). After being used in clinical trials, volunteers rated the patch favorably in terms of size, appearance, taste, and experience. Patients treated with clobetasol patch (Rivelin-CLO) experienced a significant reduction in ulcer size compared to the control group. However, most oral electrostatic spun patches are currently in the research phase. *In vivo* testing of electrostatically spun patches has been conducted primarily in animal models, and few have been tested on human oral mucosa. However, some electrostatically spun fiber films have shown good adhesion properties on isolated porcine mucosa (Zhou et al., 2022).

**TABLE 1:** Clinical application of antimicrobial dressings.

References	Antimicrobial agents	Wound dressing	Wound model	Clinical effect
Alven et al. (2022)	Silicone	Foams	Diabetic ulcers	Wound closure and reduction
Coutts and Sibbald (2014)	Gentian violet and methylene blue	PVA foams	Diabetic foot ulcers	Increased Superficial bacterial burden
Zhou et al. (2022)	Polyvinylpyrrolidone	A bilayer membrane	Common wound	Reduced ulcer area
Li et al. (2016)	Silicone	Foam	Hospital-acquired pressure injuries	Reduce injury incidence
Sharma et al. (2017)	—	Hydrofiber dressings	After total hip and knee arthroplasty	Reduce complications

A randomized controlled trial conducted in the United States reported that the use of a soft silicone foam dressing in combination with standard preventive care was statistically and clinically significant in reducing the incidence and severity of hospital-acquired pressure injuries (HAPI) in intensive care patients, compared with patients who received standard preventive care (Li et al., 2016). The results of a systematic evaluation and meta-analysis of optimal wound dressing materials after total hip and knee arthroplasty showed that wounds treated with hydrofiber dressings were significantly less likely to experience wound complications than wounds treated with passive dressings (Sharma et al., 2017).

There are only a few antimicrobial agents that can be purchased, and research indicates that wound dressings containing antimicrobials, such as silver and iodine, may be useful in avoiding or treating minor infections. (Lipsky et al., 2016). Nevertheless, the available data are insufficient to endorse the use of topical antimicrobial treatments. Researchers are actively seeking the most effective and reliable treatment approach that not only enhances and expedites the healing process, but also mitigates the risk of potential infections. (Markiewicz-Gospodarek et al., 2022).

## 5 Conclusion and perspective

Effective management of wound infection is essential for promoting wound healing, and the selection of dressings and antimicrobial therapy plays a crucial role in treating chronic wounds. In this discussion, we focus on the current use of antimicrobial wound dressings with various structures and advancements in research on different antimicrobial agents to facilitate faster wound recovery. Antibacterial dressings demonstrate strong antibacterial activity and have wide-ranging potential applications in the treatment of wound healing. Despite extensive research on dressings and antimicrobial agents, current dressings are mainly tested in animal experiments, and many antimicrobial hydrogels are only evaluated against specific bacteria such as *Staphylococcus aureus*; therefore, more experiments are needed to establish a standard for clinical application. In the field of antimicrobial materials, there are thousands of known antibiotics, but only about 1% of them are used clinically due to concerns about their side effects. Issues related to resistance to commonly used antibiotics and regulatory control over antibiotic distribution remain significant concerns. Therefore, integrating and leveraging the antimicrobial properties, biocompatibility, and mechanical properties of wound dressings to develop fully functional advanced wound dressings remains a major challenge for researchers. Looking ahead into the future with rapid advances in artificial intelligence and machine learning techniques, combining computer simulation with data analysis may enable us to predict antibiotic performance more accurately, thereby accelerating the development and optimization of new dressings.
